# Quantitation of eumelanin and pheomelanin markers in diverse biological samples by HPLC-UV-MS following solid-phase extraction

**DOI:** 10.1371/journal.pone.0223552

**Published:** 2019-10-17

**Authors:** Susanne Affenzeller, Holm Frauendorf, Tobias Licha, Daniel J. Jackson, Klaus Wolkenstein

**Affiliations:** 1 Department of Geobiology, Georg-August University, Göttingen, Germany; 2 Institute of Organic & Biomolecular Chemistry, Georg-August University, Göttingen, Germany; 3 Department of Applied Geology, Georg-August University, Göttingen, Germany; Fisheries and Oceans Canada, CANADA

## Abstract

Eumelanin and pheomelanin are well known and common pigments found in nature. However, their complex polymer structure and high thermostability complicate their direct chemical identification. A widely used analytical method is indirect determination using HPLC with UV detection of both types of melanin by their most abundant oxidation products: pyrrole-2,3-dicarboxylic acid (PDCA), pyrrole-2,3,5-tricarboxylic acid (PTCA), thiazole-4,5-dicarboxylic acid (TDCA), and thiazole-2,4,5-tricarboxylic acid (TTCA). An increasing interest in pigmentation in biological research led us to develop a highly sensitive and selective method to identify and quantify these melanin markers in diverse biological samples with complex matrices. By introducing solid-phase extraction (SPE, reversed-phase) following alkaline oxidation we could significantly decrease background signals while maintaining recoveries greater than 70%. Our HPLC-UV-MS method allows for confident peak identification via exact mass information in corresponding UV signals used for quantitation. In addition to synthetic melanin and *Sepia officinalis* ink as reference compounds eumelanin markers were detected in brown human hair and a brown bivalve shell (*Mytilus edulis*). Brown feathers from the common chicken (*Gallus g*. *domesticus*) yielded all four eumelanin and pheomelanin markers. The present method can be easily adapted for a wide range of future studies on biological samples with unknown melanin content.

## Introduction

**Fig 1:**
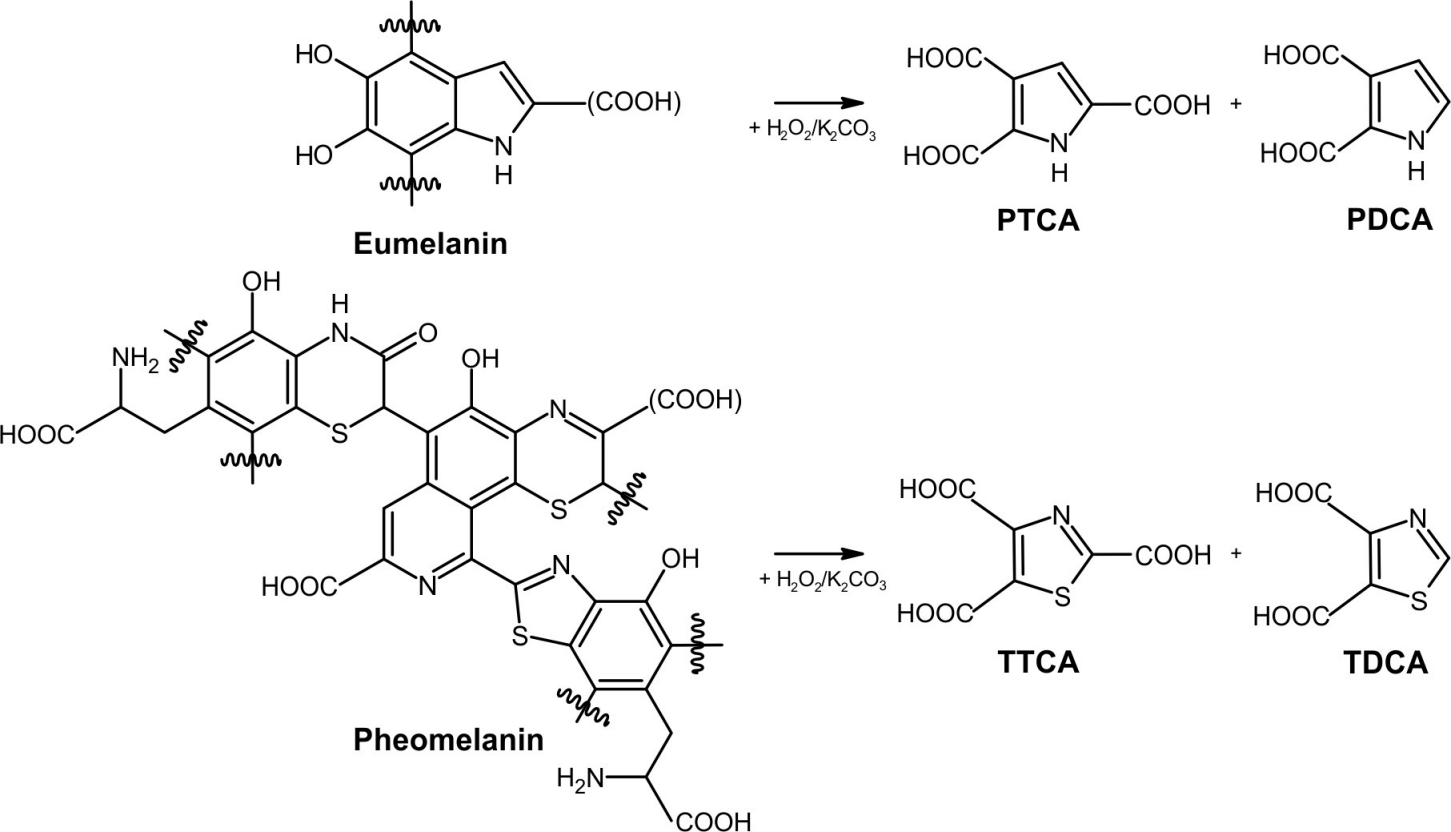
Products of eumelanin and pheomelanin generated by alkaline oxidation.

In the scientific literature the term ‘melanin’ has been used for any number of black, dark brown to orange and yellow pigments that are non-soluble and very thermostable [1–7]. A more accurate definition of melanin would be that they are built through enzymatic oxidative polymerisation of DOPA (L-3,4-dihydroxyphenylalanine) subunits [4, 8–12]. The stability and sizes of the resulting macromolecules complicate their analysis by standard analytical methods [10, 12, 13]. A well-established method in human medical studies to identify the most common melanin types (eumelanin and pheomelanin) uses the oxidative degradation products of melanin (Fig 1): pyrrole-2,3-dicarboxylic acid (PDCA), pyrrole-2,3,5-tricarboxylic acid (PTCA), thiazole-4,5-dicarboxylic acid (TDCA) and thiazole-2,4,5-tricarboxylic acid (TTCA) [6, 14–17].

Further experiments based on the initially established method of potassium permanganate as an oxidative agent [14–16] yielded a simplified and more reproducible oxidation protocol using alkaline oxidation by hydrogen peroxide [6, 18–20]. High-performance liquid chromatography (HPLC) with UV detection and a mobile phase consisting of methanol–phosphate buffer (pH 2.1) has been used to separate and detect these specific melanin oxidation products [6, 20]. This method was mainly developed to analyse human melanin in medical applications like hair and skin samples, where the presence of melanin is indisputable and interest lies mainly in quantitative analysis. But in recent years, biologists have been increasingly interested in pigments and colour patterns and their presence in a variety of biological settings [21–27]. For these biological samples with unknown pigment content a more specific method is needed. Furthermore, in biological samples like hair, feathers and shells, analyses of melanin are greatly hindered by the presence of complex organic matrices resulting from the oxidation of proteins and other compounds by H_2_O_2_. A consequence of the naturally high background signals in these biological samples is that reliable peak identification can be very difficult (compare chromatograms in Ito et al. [20], Williams et al. [25]). The introduction of a simple sample preparation step that minimizes background signals is therefore necessary. Yu et al. [28] used a liquid-liquid extraction method for this purpose, but did not systematically test the effect of this step on established melanin oxidation markers. While Rioux et al. [29] investigated the effects of solid-phase extraction (SPE) using weak anion exchange columns on melanin oxidation products, but focused solely on melanoma cells as a biological sample and refrained from adapting the method for compatibility to mass spectrometric (MS) methods.

In order to overcome the limitation of low selectivity afforded by UV detection, recent methods have replaced the phosphate buffer in the eluent with formic acid to allow MS detection. For time-of-flight (TOF) MS detection of eumelanin pigments, preparative separation of the analytes prior to MS analysis was required [23], whereas more sensitive HPLC-MS/MS methods for the analysis of the eumelanin markers PDCA and PTCA in bivalve tissue [28] and for PTCA in hair samples [30] have been reported. However, to date, no method for the full chromatographic separation and sensitive MS detection of eumelanin and pheomelanin markers has been reported.

The aim of the present study is to establish a reliable analytical method for SPE sample clean-up and subsequent detection and quantitation even of trace amounts of all four common eumelanin and pheomelanin markers from diverse biological samples.

## Materials and methods

### Chemicals and reagents

Water (HPLC gradient grade) was purchased from J.T. Baker (Deventer, The Netherlands). Pestinorm® Supra Trace ethyl acetate was purchased from VWR Chemicals (Leuven, Belgium). LiChrosolv® methanol (hypergrade for LC-MS), potassium carbonate (pro analysi grade) and calcium carbonate (pro analysi grade) were obtained from Merck KGaA (Darmstadt, Germany). Sodium sulphite (puriss p.a., ACS grade) and hydrogen peroxide solution (≥ 30%, trace analysis grade) were obtained from Sigma-Aldrich (St. Louis, USA). Rotipuran® formic acid (≥98%, p.a., ACS grade), Rotipuran® hydrochloric acid (37%, p.a., ACS, ISO grade) and Pufferan® TRIS hydrochloride (≥ 99%, p.a. grade) were purchased from Carl Roth GmbH + Co. KG (Karlsruhe, Germany). Proteinase K was purchased from Qiagen GmbH (Hilden, Germany).

### Standards and samples

Standards of the melanin oxidation products PDCA, PTCA, TDCA and TTCA (prepared according to previously published protocols [31]) were kindly provided by Prof. Shosuke Ito at stock solution concentrations of 100 μg/mL. Synthetic melanin (prepared by oxidation of tyrosine with hydrogen peroxide) and melanin from *Sepia officinalis* were obtained from Sigma-Aldrich (St. Louis, USA) and were used for method development as known eumelanin standards.

The human hair sample was donated by one of the authors (S.A.). Medium brown hair was cut approximately 10 cm from the scalp and then cut into 5 mm pieces. A brown chicken feather was obtained from a domestic chicken (*Gallus gallus domesticus*). Analyses were carried out on the distal tip of the brown feather. A shell of the bivalve *Mytilus edulis* was commercially obtained from a food market. Shell samples were taken from the distal growing edge which possessed brown longitudinal stripes.

### Sample preparation and melanin oxidation

All biological samples were cleaned with deionized water in an ultrasonic bath for 10 min and allowed to dry. For synthetic melanin and *Sepia* ink melanin 0.2 mg, for feather and hair samples 5.5 mg and for shell sample 1.5 g were used, respectively. Melanin oxidation was carried out as previously published [20, 25] with some modifications: Prepared shell pieces were dissolved in HCl (6 M, approximately 7 mL) and centrifuged at 13,000 rpm for 15 min. The obtained supernatant was discarded and the residue was washed twice with H_2_O.

Biological samples (hair, feather and shell) were treated with 10 μL proteinase K (10 mg/mL) in 500 μL TRIS-HCl buffer (1 M, pH 8.0) for 30 min at 37°C in a shaker. Treatment was stopped by the addition of 300 μL HCl (6 M). Samples were centrifuged at 13,000 rpm for 15 min, the supernatant discarded and the pellet was washed in water.

All oxidation reactions (synthetic melanin, *Sepia* ink melanin, feather, hair and shell) were carried out for 20 h at 25°C with vigorous shaking using 100 μL H_2_O, 375 μL K_2_CO_3_ (1 M) and 25 μL H_2_O_2_ (30%) as reactants. After this time any remaining H_2_O_2_ was inactivated by the addition of 50 μL Na_2_SO_3_ (10% (w/v) and 140 μL HCl (6 M). Samples were then centrifuged at 13,000 rpm for 30 min and the supernatant was transferred into a fresh tube. As a negative control 2.0 g of calcium carbonate was treated like the shell sample. For comparison, a mixture of PTCA, PDCA, TTCA and TDCA in H_2_O (2.5 μg/mL each) was run under the same conditions as the oxidised samples.

### Sample treatment using SPE

Oxidised samples were treated by SPE on Strata^™^-X 33 μm Polymeric Reversed Phase cartridges 200 mg/6 mL (Phenomenex, Torrance, USA) under vacuum. Cartridges were conditioned with 5 mL methanol followed by 5 mL H_2_O. Samples were loaded onto the SPE cartridges diluted in 5 ml formic acid (0.3% (v/v)) and washed once with 5 mL formic acid (0.3% (v/v)). The cartridges were then dried for 30 min and elution was carried out with 3 mL methanol followed by 3 mL ethyl acetate. Solvents were removed under a constant nitrogen stream at 40°C and samples were re-dissolved in 200 μL H_2_O.

### Chromatographic separation with UV and MS detection

Measurements were carried out on a Thermo Fisher Scientific HPLC-MS system consisting of an Accela HPLC with a Finnigan Surveyor PDA Detector and coupled to an LTQ Orbitrap XL mass spectrometer equipped with an electrospray ionization (ESI) source. Chromatographic separation was carried out on a Gemini C18 column (5 μm particle size, 250 × 2 mm i.d. (Phenomenex, Torrance, USA)). Aliquots (10 μL) of the samples were injected into the HPLC system operating at a flow rate of 0.2 mL/min. The mobile phase consisted of 0.3% formic acid (eluent A) and methanol (eluent B) (80:20) was run at 45°C for 20 min isocratically, followed by a wash step of A:B (5:95) for 10 min and an equilibration phase to reach starting conditions for 10 min. UV data were recorded between 200 and 400 nm. Quantitation was conducted in the range of 250–290 nm. Mass spectra were acquired in negative ion mode. The scan window was set to *m*/*z* = 120–220. Optimized MS conditions included: gas flow rate of 50 (arbitrary units), a spray voltage of 5.0 kV and a heated capillary temperature of 275°C.

### Calibration and validation

The linear range of the method was tested for each of the melanin oxidation products with a 9-point calibration curve at concentrations ranging from 0.01 to 10 μg/mL by multiple injections. Limit of detection (LOD) and limit of quantitation (LOQ) were determined with the signal-to-noise ratio method for each of the standards based on HPLC measurements with UV detection. LOD was set at 3:1 and LOQ at 10:1 signal-to-noise, respectively.

Recoveries after sample preparation by SPE were tested with a mixture of all four melanin oxidation products in eluent A. Additionally, total method recovery was investigated for all three natural matrices (feather, hair, shell) by a 3-point standard addition (2 times, 5 times and 10 times) of all oxidation products. Standards were added following oxidation of matrices and prior to SPE. SPE recoveries without matrices were measured on an Agilent 1200 Series HPLC system with diode array detector using the same chromatographic conditions as described above.

Additional experiments on the oxidation protocol itself verified the linearity of PDCA and PTCA formation from a synthetic eumelanin standard in the range of 0.05–0.4 mg. A test with an elongated oxidation time (40 h) did not result in significantly higher amounts of oxidation products and even yielded slightly less eumelanin markers in the case of shell samples.

## Results and discussion

### Method development

Our method for alkaline oxidation of melanin from a wide range of biological samples combines and refines a variety of previously published protocols [20, 21, 23, 25]. We demonstrate that by including a sample clean-up step by SPE and adapting the chromatographic system to allow for dual detection with UV and MS, two markers each for eumelanin (PDCA and PTCA) and pheomelanin (TDCA and TTCA) can be analysed within one HPLC run. We could achieve baseline separation of all four melanin oxidation markers for chromatographic conditions compatible with mass spectrometry. By desalting and purifying samples via SPE we could significantly reduce the background of the diverse samples we investigated. In addition, the evaporation step brings the advantage of concentrating the analytes. A surprising observation during reversed-phase HPLC optimization was that the retention time of the analytes was almost unaffected by the concentration of organic solvent (15 to 25% methanol in the eluent). We therefore used isocratic instead of gradient elution. Instead, retention times were strongly affected by the pH value of the eluent, an effect also observed in a recent study [29].

**Fig 2:**
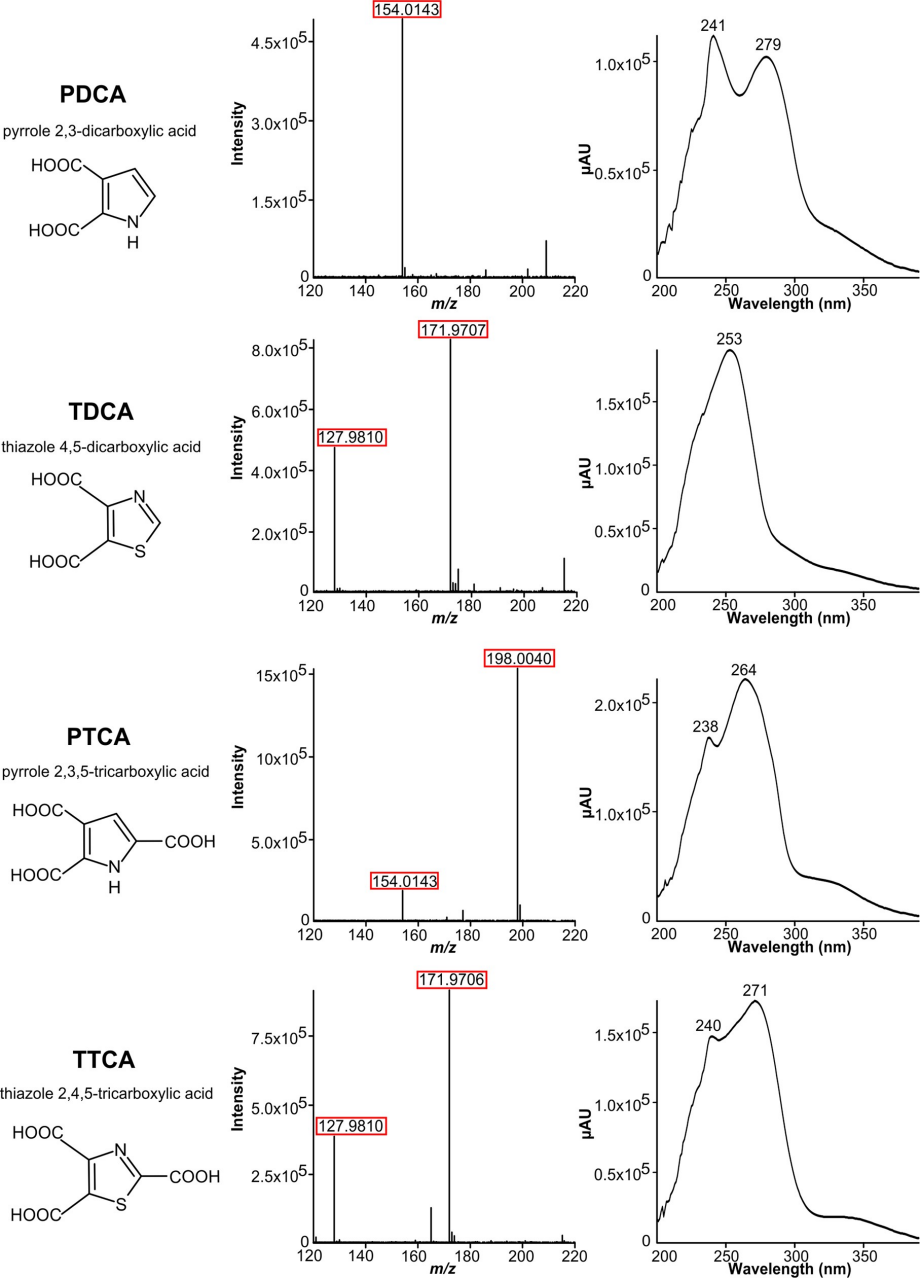
Characterization of the melanin oxidation products PDCA, TDCA, PTCA and TTCA from brown chicken feather by mass spectra (negative ion mode) and UV spectra.

**Fig 3:**
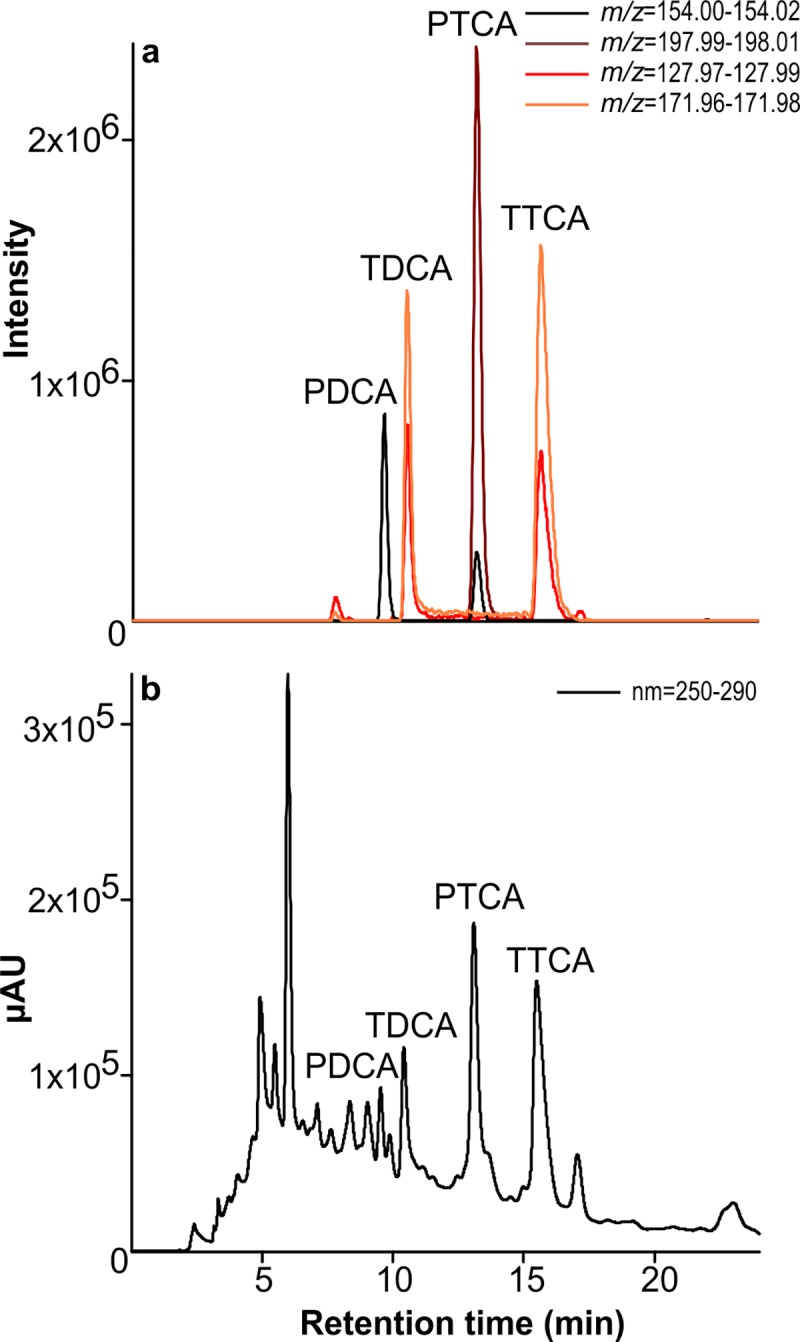
Comparison of extracted ion chromatograms. (**a**) and corresponding UV chromatogram (**b**) of oxidation products from a brown chicken feather following SPE, highlighting the need for peak confirmation in biological samples.

The high-resolution ESI mass spectra of PDCA, TDCA and PTCA show ion signals for the deprotonated molecules [M–H]^–^. TDCA and PTCA show additional ions resulting from fragmentation of CO_2_ from one of the carboxyl groups. In contrast, for TTCA only fragment ions resulting from the loss of one and two carboxyl groups can be observed, yielding virtually the same mass spectrum as TDCA (see Table 1 and Fig 2). Therefore, the identification and quantitation of both TDCA and TTCA is only possible by the inclusion of a chromatographic separation. All four melanin oxidation products can be characterized by their specific UV absorption spectra (Fig 2), when there are no interfering background peaks. Quantitation in MS requires appropriate internal standards which are not commercially available for the analysed melanin markers. In contrast, quantitation with UV detection can be done with melanin oxidation product standards via external calibration or standard addition. However, peak identification must be carried out very carefully due to the naturally high backgrounds present in biological samples even after clean-up by SPE (compare Fig 3). By coupling UV with high-resolution MS detection, straight-forward quantitation based on UV signals can be combined with reliable compound identification in complex biological samples. In addition, mass spectrometric measurements yield an approximately 1.5 times higher sensitivity than UV detection, an important factor when analysing biological samples with unknown melanin content.

**Table 1:** Accurate mass data of melanin oxidation products.

Compound	Molecular formula	Calculated [M–H]^−^*m*/*z*	Observed [M–H]^−^*m*/*z*	Observed fragment ions *m*/*z*
PDCA	C_6_H_5_NO_4_	154.0140	154.0143	–
PTCA	C_7_H_5_NO_6_	198.0039	198.0040	154.0143
TDCA	C_5_H_3_NO_4_S	171.9705	171.9706	127.9810
TTCA	C_6_H_3_NO_6_S	215.9603	–	171.9706, 127.9810

Previous method validations for the analyses of melanin oxidation products have not been thoroughly performed [6, 17, 20, 32]. We present here calibration and validation data for the quantitation of all four melanin oxidation product standards via UV detection (Table 2) following SPE. Linearity could be shown for both eumelanin oxidation markers (PDCA and PTCA) in the range from 0.05–10 μg/mL. For pheomelanin oxidation markers (TDCA and TTCA) linearity ranged from 0.1–10 μg/mL.

**Table 2:** Limit of detection (LOD), limit of quantitation (LOQ) and linearity (R^2^) for HPLC with UV detection.

Compound	LOD (μg/mL)	LOQ (μg/mL)	Linearity (R^2^)	Range of linearity (μg/mL)
PDCA	0.03	0.08	0.995	0.05–10
PTCA	0.04	0.10	0.994	0.05–10
TDCA	0.08	0.25	0.994	0.1–10
TTCA	0.10	0.33	0.990	0.1–10

A recently published HPLC-MS/MS study on human hair determined LOD and LOQ for one of the four standard melanin oxidation markers (PTCA) [30]. Our method allows all four oxidation products to be detected in concentrations of 0.1 μg/mL or less, for PTCA below or at a comparable level to LODs previously published [21, 29, 30]. Both eumelanin markers can be quantified at concentrations as low as 0.1 μg/mL. For pheomelanin markers the lowest quantifiable concentrations were 0.25 μg/mL for TDCA and 0.33 μg/mL for TTCA.

Recovery of melanin oxidation products in eluent A after SPE ranged from 67% for TTCA to 95% for TDCA (Table 3). A comparison of quantitation by external calibration and standard addition in all three tested biological matrices yielded good results with more than 70% recovery for the eumelanin markers PDCA and PTCA. The pheomelanin markers TDCA and TTCA display a wide range of recoveries from different matrices. This might be explained by the interactions of different matrices on the SPE conditions as well as the purity of the peaks. These observations emphasize that accurate and precise quantitation of pheomelanin markers in biological matrices must be done very carefully with the appropriate controls and replicates, and any results should be interpreted critically.

**Table 3:** Effect of biological matrices on the recovery of melanin oxidation products following SPE.

Compound	Recovery (%) ± SD			
	No matrix^a^	Feather^b^	Hair^b^	Shell^b^
PDCA	82 ± 1^c^	93 ± 11^c^	71	81
PTCA	76 ± 4^c^	87 ± 9^c^	89	81
TDCA	95 ± 4^c^	179 ± 45^c^	64	194
TTCA	67 ± 8^c^	52 ± 5^c^	114	69

^a^ Values determined on an Agilent 1200 Series HPLC system

^b^ Three level standard additions following oxidation and prior to SPE

^c^ Values were calculated as means of n = 3

### Application of method to complex biological samples

**Fig 4:**
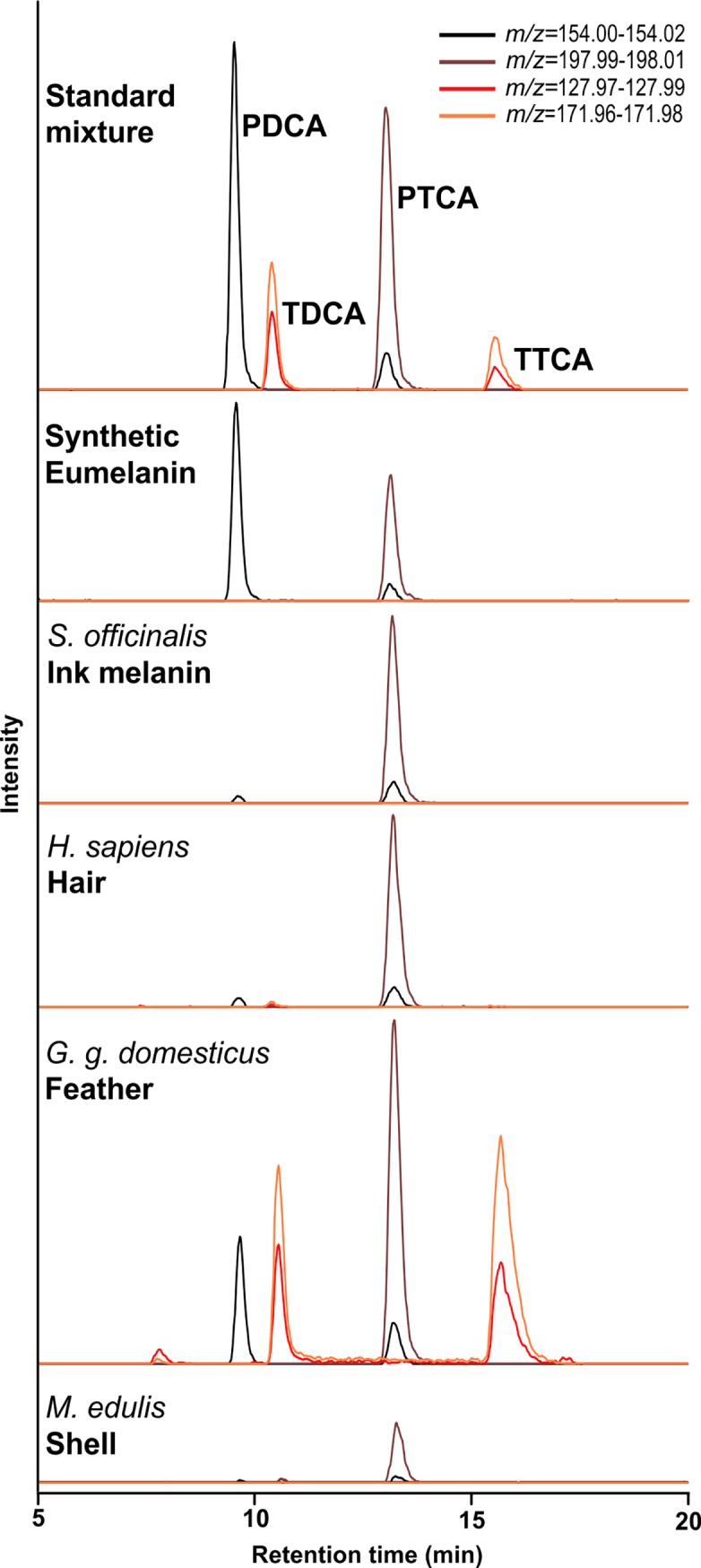
Extracted ion chromatograms of melanin oxidation product standards and melanin reference compounds (standard mixture of oxidation products, synthetic eumelanin, *Sepia* ink melanin) as well as biological samples (human hair, chicken feather, bivalve shell) following alkaline oxidation.

Both eumelanin markers PDCA and PTCA were found in synthetic melanin and *Sepia officinalis* ink melanin (Fig 4). In synthetic melanin both eumelanin oxidation products can be found in similar amounts (PDCA: 55.6% and PTCA 44.4% of total melanin markers), whereas in *S*. *officinalis* ink PTCA is the predominant marker (94.8% of total melanin markers), with only small amounts of PDCA (5.2% of total melanin markers) (Table 4). This effect of differing ratios of oxidation product yields leads to the assumption of different compositions of the parent macromolecules [14, 20, 31]. Each biological sample investigated in this study produced a different PTCA/PDCA ratio (Table 4). Further research on the polymerisation process of natural melanins and investigations on their chemical structure would be needed to understand these differences and their effects on colour and functionality. As expected, pheomelanin oxidation products TDCA and TTCA could not be detected in either of the melanin reference compounds measured here.

**Table 4:** Amounts of melanin oxidation products in melanin standards and biological samples per g of oxidised starting material as quantified by HPLC with UV detection.

Sample	Eumelanin markers		Pheomelanin markers	
	PDCA (μg/g sample)	PTCA (μg/g sample)	TDCA (μg/g sample)	TTCA (μg/g sample)
Synthetic melanin	1206.23	963.80	< LOD	< LOD
*S*. *officinalis* ink melanin	99.40	1808.25	< LOD	< LOD
*H*. *sapiens* brown hair	3.75	60.60	< LOQ	< LOD
*G*. *g*. *domesticus* brown feather	43.25	119.92	108.99	403.73
*M*. *edulis* brown shell	0.03	0.11	< LOD	< LOD

In all three of the investigated biological matrices (feather, hair, shell) we were able to detect eumelanin (Fig 4 and Table 4). In the feather and hair samples we could also detect pheomelanin. It has been shown already that feathers of North American barn swallows (*Hirundo rustica erythrogaster*) contain both eumelanins and pheomelanins [21]. The same study investigated yellow chicken plumage from nestlings and found trace amounts of eumelanin. In applying our method to adult brown chicken feathers we detected an abundance of all four oxidation products, confirming the results of previous electron spin resonance investigations [33].

A difficult biological matrix from which to extract organic macromolecules are the calcified shells of molluscs. The pigment bearing layer of *Mytilus edulis* is very thin, providing only small amounts of pigment from relatively large amounts of shell material. Nonetheless, we were able to detect eumelanin in this bivalve, providing further evidence that our method is sensitive enough to detect these pigments in a range of biological matrices. Pigmentation and the use of melanin to pattern shells and nacreous materials by a variety of molluscs have seen an increase in research in recent years [28, 34, 35]. Our method facilitates working with these complex samples and will hopefully lead to further investigations in melanic mollusc shell pigmentation.

An especially challenging sample type consists of fossilized tissues and matrices containing melanin. Although melanin seems to fossilize very well [23, 36, 37] only few researchers have access to enough material to analyse these samples with chromatographic methods with UV detection. Mass spectrometric measurements were previously performed for fossilized *Sepia* ink [23] which found evidence for eumelanin. The protocols presented here are a good starting point for further adjustments of sample preparation with SPE, and the development of even more sensitive MS methods for fossil samples suspected to contain melanin. Preliminary measurements using MS/MS detection have shown that the sensitivity of our method can be further improved by several orders of magnitude.

## Conclusion

The method we present here allows researchers to detect eumelanin and pheomelanin in a variety of complex biological samples. The cleaning and concentrating effect afforded by SPE and the addition of mass spectrometry allows for the selective identification of even small amounts (0.1 μg/mL or less) of known melanin oxidation products. High-resolution mass spectrometry allows confident peak identification even in complex biological samples with interfering background and overlapping peaks in chromatograms provided by UV detection.

In contrast to the difficult analysis of their parent macromolecules, oxidation products for eumelanin and pheomelanin can be quantitated with the present method. However, due to the different compositions of natural melanins (e.g. PDCA/PTCA ratio differences in reference compounds and biological samples) and matrix effects of biological samples on SPE (see Table 3), we recommend the use of both oxidation markers for each type of melanin as indicators for the abundance of eumelanin and pheomelanin pigments in the original sample.

The highly sensitive method that we report here improves our ability to simultaneously detect eumelanin and pheomelanin in a variety of complex biological samples.
